# Transcatheter Aortic Valve Replacement in Patients With or Without Active Cancer

**DOI:** 10.1161/JAHA.123.030072

**Published:** 2023-10-27

**Authors:** Tadao Aikawa, Toshiki Kuno, Aaqib H. Malik, Alexandros Briasoulis, Dhaval Kolte, Polydoros N. Kampaktsis, Azeem Latib

**Affiliations:** ^1^ Department of Cardiology Juntendo University Urayasu Hospital Urayasu Japan; ^2^ Department of Radiology Jichi Medical University Saitama Medical Center Saitama Japan; ^3^ Division of Cardiology, Montefiore Medical Center Albert Einstein College of Medicine New York NY USA; ^4^ Division of Cardiology, Jacobi Medical Center Albert Einstein College of Medicine New York NY USA; ^5^ Department of Cardiology Westchester Medical Center Valhalla NY USA; ^6^ Division of Cardiovascular Medicine, Section of Heart Failure and Transplantation University of Iowa Iowa City IA USA; ^7^ Division of Cardiology Massachusetts General Hospital and Harvard Medical School Boston MA USA; ^8^ Division of Cardiology Columbia University Irving Medical Center New York NY USA

**Keywords:** active cancer, bleeding, mortality, readmission, transcatheter aortic valve replacement, Catheter-Based Coronary and Valvular Interventions

## Abstract

**Background:**

Data on clinical outcomes after transcatheter aortic valve replacement (TAVR) in specific cancer types or the presence of metastatic disease remain sparse. This study aimed to investigate the impact of active cancer on short‐term mortality, complications, and readmission rates after TAVR across different cancer types.

**Methods and Results:**

The authors assessed the Nationwide Readmissions Database for TAVR cases from 2012 to 2019. Patients were stratified by specific cancer types. Primary outcome was in‐hospital mortality. Secondary outcomes included bleeding requiring blood transfusion and readmissions at 30, 90, and 180 days after TAVR. Overall, 122 573 patients undergoing TAVR were included in the analysis, of whom 8013 (6.5%) had active cancer. After adjusting for potential confounders, the presence of active cancer was not associated with increased in‐hospital mortality (adjusted odds ratio [aOR], 1.06 [95% CI, 0.89–1.27]; *P*=0.523). However, active cancer was associated with an increased risk of readmission at 30, 90, and 180 days after TAVR and increased risk of bleeding requiring transfusion at 30 days. Active colon and any type of metastatic cancer were individually associated with readmissions at 30, 90, and 180 days after TAVR. At 30 days after TAVR, colon (aOR, 2.51 [95% CI, 1.68–3.76]; *P*<0.001), prostate (aOR, 1.40 [95% CI, 1.05–1.86]; *P*=0.021), and any type of metastatic cancer (aOR, 1.65 [95% CI, 1.23–2.22]; *P*=0.001) were individually associated with an increased risk of bleeding requiring transfusion.

**Conclusions:**

Patients with active cancer had similar in‐hospital mortality after TAVR but higher risk of readmission and bleeding requiring transfusion, the latter depending on certain types of cancer.

**Nonstandard Abbreviations and Acronyms**
AS: aortic stenosis
NRD: Nationwide Readmissions Database
PPM: permanent pacemaker
TAVR: transcatheter aortic valve replacement

**Clinical Perspective**

**What Is New?**

- Active cancer was not associated with increased in‐hospital mortality but was associated with an increased risk of readmission at 30, 90, and 180 days after transcatheter aortic valve replacement (TAVR) and increased risk of bleeding requiring transfusion at 30 days after TAVR.
- Among individual active cancer types, active colon cancer and any type of metastatic cancer were individually associated with readmissions at 30, 90, and 180 days after TAVR and an increased risk of bleeding requiring transfusion at 30 days after TAVR.

**What Are the Clinical Implications?**

- TAVR may be a treatment option for certain patients with active cancer and severe aortic stenosis. An individualized approach to these patients is needed to determine the benefit of TAVR and dictate personalized treatment strategies based on the types and extent of cancers.

Cardiovascular disease and cancer are the 2 leading causes of death in the United States and tend to coexist in the elderly population.^1^ In general, concomitant active cancer is considered a barrier to valve replacement in patients with severe aortic stenosis (AS) due to the limited life expectancy of these patients.^2^ Current evidence on outcomes after transcatheter aortic valve replacement (TAVR) in patients with active cancer is relatively limited, because pivotal randomized trials of TAVR excluded patients with a limited life expectancy.^3^, ^4^ In practice, however, the outcomes of patients with cancer can be difficult to predict, and TAVR may offer these patients a chance to be effectively treated for cancer, particularly with reduced postprocedural acute kidney injury and major bleeding compared with surgical aortic valve replacement.^5^ Although patients with active cancer and severe AS could be considered for TAVR, there is evidence to suggest a higher risk of bleeding complications after TAVR in these patients.^6^, ^7^ Among various cancers, colon cancer may be associated with a significant risk of postprocedural bleeding after TAVR, as with percutaneous coronary intervention for acute myocardial infarction.^8^ On the other hand, active cancer is known to increase thrombotic events given its hypercoagulable state.^8^ Recent retrospective studies have suggested no significant differences in all‐cause mortality at 30 days between patients with or without active cancer.^7^, ^9^ However, data on clinical outcomes after TAVR in specific cancer types or the presence of metastatic disease remain sparse. This study aimed to investigate the impact of active cancer on short‐term mortality, complications, and midterm readmissions after TAVR across different cancer types.

## METHODS

This study was exempted from institutional review board approval or written informed consent owing to the use of publicly available deidentified data. The data that support the findings of this study are available from the corresponding author upon reasonable request.

### Study Design, Population, and Outcomes

We queried the Nationwide Readmissions Database (NRD), which is the largest publicly available all‐payer database developed for the Healthcare Cost and Utilization Project in the United States. The NRD contains data from roughly 18 million discharges and 35 million weighted discharges each year, accounting for 62% of all US hospitalizations.^10^, ^11^, ^12^, ^13^, ^14^, ^15^, ^16^

The study cohort included patients ≥18 years of age undergoing TAVR between 2012 and 2019 who were discharged from January to November, because the NRD does not track patients across calendar years. The primary outcome of interest for this study was in‐hospital mortality. Secondary outcomes included acute kidney injury, acute kidney injury requiring dialysis, bleeding requiring blood transfusion, permanent pacemaker (PPM) implantation, stroke, and readmissions at 30, 90, and 180 days post‐TAVR. The 90‐ and 180‐day readmission rates were calculated after excluding patients who were discharged from October to December and those from July to December, respectively, because 90 and 180 days of follow‐up would not be available for these patients.

TAVR cases were identified using *International Classification of Diseases, Ninth Revision and Tenth Revision, Clinical Modification* (*ICD‐9‐CM* and *ICD‐10‐CM*) codes 35.05 and 35.06 for *ICD‐9‐CM* or codes 02RF37H, 02RF37Z, 02RF38H, 02RF38Z, 02RF3JH, 02RF3JZ, 02RF3KH, and 02RF3KZ for *ICD‐10‐CM*.^12^, ^17^, ^18^ Active cancer status was identified using *Clinical Classification Software* codes 11 to 41 based on the previous studies from the NRD (Table S1).^8^

### Statistical Analysis

Differences between the groups (no active cancer versus each type of cancer) were assessed by 1‐way ANOVA for continuous variables and the χ^2^ test for categorical variables. The normality assumption of age was tested by the Kolmogorov‐Smirnov test. A multilevel logistic regression analysis accounting for strata and hospital clustering was performed to examine the association between active cancer status and outcomes. Furthermore, the relative effects of specific cancer types and the presence or absence of any type of metastatic cancers on outcomes were evaluated including colon cancer, lung cancer, prostate cancer, and breast cancer, which are the 4 most common cancers in the United States.^5^, ^19^ Patient demographics and comorbidities such as age, sex, congestive heart failure, hypertension, diabetes, obesity, peripheral vascular disease, coronary artery disease, atrial fibrillation, chronic pulmonary disease, pulmonary circulation disorders, other neurological disorders, hypothyroidism, chronic kidney disease or end‐stage renal disease, liver disease, anemia, rheumatic disease, coagulopathy, abnormal weight loss, prior percutaneous coronary intervention, prior coronary artery bypass grafting, prior myocardial infarction, prior stroke, primary payer, median household income, and hospital characteristics including hospital bed size, location, and teaching status listed in Table 1 were included as covariates for multivariable analysis. Coding algorithms of *ICD‐9‐CM* and *ICD‐10‐CM* for defining comorbidities were based on the well‐validated methodology.^20^ Hospital characteristics were defined as previously described.^10^ This database has a variable DISCWT, which is the discharge‐level weight on the Healthcare Cost and Utilization Project databases and is used for producing the national estimates. SURVEY or SVY commands were used to analyze the data in accordance with specific analytic considerations for publicly accessible large data sets.^21^ Coding algorithms of *ICD‐9‐CM* and *ICD‐10‐CM* for defining clinical events were based on previous studies,^8^, ^22^ as shown in Table S2. We excluded all the missing variables and performed a complete case analysis.

**Table 1:** Baseline Characteristics of Patients With and Without Active Cancer Who Underwent Transcatheter Aortic Valve Replacement

Characteristics	No cancer (n=114 560)	Any cancer (n=8013)	*P* value
Weighted population	211 436	14 716	
Age, y*	79.6±8.6	79.6±8.4	<0.001
Median (IQR)	81 (75–86)	81 (75–86)	0.156†
Women	46.8%	41.1%	<0.001
Insurance			0.077
Medicare	90.5%	89.6%	
Medicaid	1.3%	1.2%	
Private	6.1%	6.8%	
Other	2.1%	2.4%	
Median household income			<0.001
$1–$38 999	21.5%	17.8%	
$39 000–$47 999	27.4%	26.2%	
$48 000–$62 999	27.2%	27.6%	
≥$63 000	23.9%	28.4%	
Comorbidities			
Congestive heart failure	75.2%	76.8%	0.002
Hypertension	87.9%	84.2%	<0.001
Diabetes	38.4%	34.9%	<0.001
Obesity	19.6%	16.4%	<0.001
Peripheral vascular disease	24.1%	23.1%	0.087
Coronary artery disease	70.0%	66.7%	<0.001
Atrial fibrillation	40.0%	40.6%	0.326
Chronic pulmonary disease	32.4%	34.7%	<0.001
Pulmonary circulation disorders	19.7%	21.9%	<0.001
Other neurological disorders	5.7%	6.2%	0.054
Hypothyroidism	19.3%	19.9%	0.217
CKD or end‐stage renal disease	35.8%	38.9%	<0.001
Liver disease	3.6%	5.5%	<0.001
Anemia	5.0%	7.4%	<0.001
Rheumatic disease	4.8%	4.8%	0.992
Coagulopathy	14.4%	20.5%	<0.001
Abnormal weight loss	3.7%	6.4%	<0.001
Prior CABG	18.4%	14.7%	<0.001
Prior myocardial infarction	12.3%	10.3%	<0.001
Prior PCI	21.2%	18.4%	<0.001
Prior stroke	13.8%	11.2%	<0.001
Hospital characteristics			
Hospital bed size			0.001
Small	4.7%	4.0%	
Medium	20.0%	18.7%	
Large	75.3%	77.3%	
Hospital location/teaching status			<0.001
Rural teaching	10.5%	9.4%	
Urban teaching	88.6%	89.8%	
Nonteaching	0.9%	0.8%	

Values are mean±SD, %, or median (interquartile range). CABG indicates coronary artery bypass grafting; CKD, chronic kidney disease; IQR, interquartile range, and PCI, percutaneous coronary intervention.

* The normality assumption of age was not violated by the Kolmogorov‐Smirnov test (*P*=0.349).

^†^ The difference in age between the groups was assessed by Mann‐Whitney *U* test.

A sensitivity analysis was performed in the propensity score‐matched cohort to investigate the impact of any active cancer on outcomes. A propensity score was calculated by logistic regression analysis including age, sex, congestive heart failure, hypertension, diabetes, obesity, peripheral vascular disease, coronary artery disease, atrial fibrillation, chronic pulmonary disease, pulmonary circulation disorders, other neurological disorders, hypothyroidism, chronic kidney disease or end‐stage renal disease, liver disease, anemia, rheumatic disease, coagulopathy, abnormal weight loss, prior percutaneous coronary intervention, prior coronary artery bypass grafting, prior myocardial infarction, and prior stroke. Observation weights were also incorporated as the variable DISCWT in the propensity score model. Propensity score matching was performed by 1:1 nearest‐neighbor matching using a caliper width of 0.2 of the standard deviation of the propensity score, adjusting for the same covariates in the multivariable analysis. To assess the performance of the matching, standardized mean differences were calculated. Statistical analysis was performed using Stata 16.1 (StataCorp, College Station, TX) and R version 3.5.1 (R Foundation for Statistical Computing, Vienna, Austria). For all tests, a 2‐sided *P*<0.05 was considered statistically significant.

## RESULTS

Between 2012 and 2019, there were 122 573 patients undergoing TAVR who met the inclusion criteria; 114 560 without active cancer and 8013 (6.5%) with active cancer were eligible for this analysis, including 215 with colon cancer, 451 with lung cancer, 863 with prostate cancer, 382 with breast cancer, and 554 of any type of active metastatic cancer. Rates of active cancer among patients undergoing TAVR did not significantly change between 2012 and 2019 (*P* for trend=0.705).

Baseline patient characteristics are shown in Tables 1 and 2. Compared with patients without active cancer, those with active cancer were less likely to have hypertension, diabetes, obesity, coronary artery disease, and history of myocardial infarction (Table 1). Among individual active cancer types (Table 2), the majority of patients with colon and lung cancer were men. Anemia was more prevalent in patients with colon cancer (16.7%). Peripheral vascular disease and chronic pulmonary disease were more prevalent in patients with lung cancer (32.5% and 58.8%, respectively). The mean inflation‐adjusted cost of hospital stay was significantly higher in patients with colon ($60 366), lung ($59 452), and any type of active metastatic cancer ($59 610) than those without cancer ($54 770).

**Table 2:** Baseline Characteristics of Patients With and Without Specific Cancer Types

Characteristics	No cancer (n=114 560)	Colon cancer (n=215)	*P* value (vs no cancer)	Lung cancer (n=451)	*P* value (vs no cancer)	Prostate cancer (n=863)	*P* value (vs no cancer)	Breast cancer (n=382)	*P* value (vs no cancer)	Metastatic cancer (n=554)	*P* value (vs no cancer)
Weighted population	211 436	381		808		1541		693		970	
Age, y	79.6±8.6	79.4±8.2	<0.001	77.9±7.8	<0.001	81.6±6.8	<0.001	78.6±8.3	<0.001	78.4±8.6	0.002
Women	46.8%	45.0%	0.607	43.5%	0.191	0%	<0.001	96.5%	<0.001	33.4%	<0.001
Insurance			0.789		0.693		0.043		0.816		0.048
Medicare	90.5%	88.4%		89.3%		91.0%		91.2%		86.1%	
Medicaid	1.3%	1.7%		1.9%		0.2%		0.7%		2.1%	
Private	6.1%	7.5%		7.1%		5.7%		6.7%		8.5%	
Other	2.1%	2.4%		1.7%		3.1%		1.4%		3.3%	
Median household income			0.107		0.052		<0.001		0.697		0.100
$1–$38 999	21.5%	22.8%		18.1%		17.1%		18.8%		21.0%	
$39 000–$47 999	27.4%	23.9%		29.6%		23.5%		29.0%		22.5%	
$48 000–$62 999	27.2%	34.5%		23.7%		29.9%		28.1%		28.8%	
≥$63 000 or more	23.9%	18.9%		28.6%		29.5%		24.0%		27.7%	
Comorbidities											
Congestive heart failure	75.2%	70.8%	0.192	75.1%	0.986	73.1%	0.204	74.4%	0.750	74.2%	0.626
Hypertension	87.9%	86.3%	0.539	87.0%	0.566	85.1%	0.035	88.6%	0.695	85.3%	0.086
Diabetes	38.4%	44.7%	0.096	31.6%	0.006	31.1%	<0.001	38.0%	0.889	36.3%	0.367
Obesity	19.6%	15.3%	0.117	14.8%	0.011	10.6%	<0.001	25.4%	0.009	16.4%	0.080
Peripheral vascular disease	24.1%	20.1%	0.250	32.5%	<0.001	25.5%	0.419	14.6%	<0.001	21.9%	0.286
Coronary artery disease	70.0%	62.1%	0.019	66.5%	0.115	73.5%	0.060	60.0%	<0.001	66.4%	0.097
Atrial fibrillation	40.0%	33.9%	0.091	41.7%	0.514	39.1%	0.601	34.2%	0.028	37.2%	0.208
Chronic pulmonary disease	32.4%	34.7%	0.537	58.8%	<0.001	25.0%	<0.001	26.4%	0.029	27.9%	0.047
Pulmonary circulation disorders	19.7%	26.5%	0.043	15.6%	0.038	17.1%	0.095	23.3%	0.110	18.4%	0.547
Other neurological disorders	5.7%	2.7%	0.054	5.0%	0.518	4.6%	0.206	4.0%	0.190	6.2%	0.676
Hypothyroidism	19.3%	17.0%	0.475	17.5%	0.367	11.0%	<0.001	25.3%	0.006	16.7%	0.183
CKD or end‐stage renal disease	35.8%	36.2%	0.917	32.4%	0.190	35.2%	0.755	28.4%	0.005	36.6%	0.717
Liver disease	3.6%	5.6%	0.109	3.8%	0.841	3.3%	0.712	4.4%	0.375	3.4%	0.844
Anemia	5.0%	16.7%	<0.001	6.3%	0.253	5.7%	0.421	6.7%	0.216	6.8%	0.079
Rheumatic disease	4.8%	0.5%	0.003	5.3%	0.667	2.8%	0.006	4.2%	0.586	3.3%	0.097
Coagulopathy	14.4%	11.9%	0.327	14.7%	0.892	14.4%	0.979	13.1%	0.486	14.6%	0.906
Abnormal weight loss	3.7%	5.6%	0.192	4.7%	0.287	4.8%	0.134	4.7%	0.345	8.9%	<0.001
Prior CABG	18.4%	11.8%	0.032	14.3%	0.044	21.7%	0.023	5.5%	<0.001	13.2%	0.002
Prior myocardial infarction	12.3%	12.7%	0.866	11.6%	0.696	13.0%	0.562	8.3%	0.019	11.3%	0.499
Prior PCI	21.2%	13.9%	0.028	18.9%	0.253	22.0%	0.613	17.1%	0.071	14.8%	<0.001
Prior stroke	13.8%	6.6%	0.001	9.5%	0.015	11.4%	0.058	10.0%	0.054	9.4%	0.003
Hospital characteristics											
Hospital bed size			0.253		0.253		0.116		0.501		0.272
Small	4.7%	1.9%		1.9%		3.2%		3.4%		4.3%	
Medium	20.0%	21.7%		21.7%		21.5%		21.0%		17.5%	
Large	75.3%	76.4%		76.4%		75.3%		75.6%		78.2%	
Hospital location/teaching status			0.574		0.574		0.087		0.569		0.002
Rural teaching	10.5%	10.3%		10.3%		10.3%		10.2%		6.9%	
Urban teaching	88.6%	89.7%		89.7%		89.2%		88.6%		92.3%	
Nonteaching	0.9%	0%		0.0%		0.4%		1.2%		0.7%	

Values are mean±SD or %. CABG indicates coronary artery bypass grafting; CKD, chronic kidney disease; and PCI, percutaneous coronary intervention.

Crude in‐hospital mortality and the rates of readmission at 30, 90, and 180 days after TAVR were higher in patients with versus without active cancer (Table 3). In regard to TAVR‐related complications, acute kidney injury (16.6% versus 12.3%), PPM implantation (11.0% versus 9.9%), and bleeding requiring blood transfusion (14.1% versus 8.3%) were more frequent in patients with versus without active cancer. Patients with any type of active metastatic cancer were at higher risk for TAVR‐related complications than those without active cancer, such as acute kidney injury (20.0% versus 12.3%), PPM implantation (12.6% versus 9.9%), and bleeding requiring blood transfusion (12.4% versus 8.3%). Among individual active cancer types (Table 4), patients with colon cancer had a higher rate of bleeding requiring transfusion after TAVR than those without any active cancer (18.8% versus 8.3%). Patients with breast cancer had a higher rate of PPM implantation after TAVR than those without active cancer (14.5% versus 9.9%). The 30‐day readmission rate was higher in patients with active colon cancer (25.0%) or any type of active metastatic cancers (17.0%) than in those without active cancer (13.3%). The 90‐ and 180‐day readmission rates were higher for all types of active cancer except for prostate cancer. Patients with active cancer had higher rates of readmission for bleeding at 30, 90, and 180 days after TAVR (1.6%, 2.4%, and 3.1%, respectively) than those without (0.8%, 1.1%, and 1.5%, respectively; *P*<0.001 for all). Among individual active cancer types, patients with colon cancer had higher rates of readmission for bleeding at 30, 90, and 180 days after TAVR (6.5%, 9.7%, and 11.4%, respectively). The major causes of readmission at 30, 90, and 180 days after TAVR in patients with active cancer were congestive heart failure and bleeding requiring transfusion (Table 3).

**Table 3:** In‐Hospital Outcomes and Readmissions After TAVR in Patients With and Without Active Cancer

Characteristics	No cancer (n=114 560)	Any cancer (n=8013)	*P* value
In‐hospital death	2.1%	2.9%	<0.001
Discharge disposition			<0.001
Routine	58.5%	53.5%	
Skilled nursing facility	14.8%	16.8%	
Home health care	24.0%	26.4%	
AKI	12.3%	16.6%	<0.001
AKI leading to dialysis	1.0%	1.8%	<0.001
PPM implantation	9.9%	11.0%	0.003
Bleeding requiring transfusion	8.3%	14.1%	<0.001
Stroke/TIA	0.4%	0.6%	0.091
Length of hospital stay, d	3 (2–6)	3 (2–8)	<0.001
Total inflation‐adjusted cost, US$	$54 770±$31 888	$61 380±$40 208	<0.001
30‐d readmission rate	13.3%	16.8%	<0.001
Weighted population at 30 d	211 436	14 716	
Causes of readmission at 30 d			
Congestive heart failure	2.6%	3.0%	0.002
Myocardial infarction	0.2%	0.2%	0.995
Stroke/TIA	0.4%	0.4%	0.269
Bleeding requiring transfusion	0.8%	1.6%	<0.001
90‐d readmission rate	22.5%	29.8%	<0.001
Weighted population at 90 d	168 310	11 747	
Causes of readmission at 90 d			
Congestive heart failure	2.7%	2.8%	0.739
Myocardial infarction	0.3%	0.3%	0.497
Stroke/TIA	0.8%	0.6%	0.102
Bleeding requiring transfusion	1.1%	2.4%	<0.001
180‐d readmission rate	30.8%	40.1%	<0.001
Weighted population at 180 d	107 846	7435	
Causes of readmission at 180 d			
Congestive heart failure	3.5%	3.6%	0.705
Myocardial infarction	0.5%	0.4%	0.351
Stroke/TIA	1.1%	1.0%	0.263
Bleeding requiring transfusion	1.5%	3.1%	<0.001

Values are mean±SD, median (interquartile range), or %. AKI indicates acute kidney injury; PPM, permanent pacemaker; and TIA, transient ischemic attack.

**Table 4:** In‐Hospital Outcomes and Readmissions After TAVR in Patients With and Without Specific Cancer Types

Characteristics	No cancer (n=114 560)	Colon cancer (n=215)	*P* value (vs no cancer)	Lung cancer (n=451)	*P* value (vs no cancer)	Prostate cancer (n=863)	*P* value (vs no cancer)	Breast cancer (n=382)	*P* value (vs no cancer)	Metastatic cancer (n=554)	*P* value (vs no cancer)
In‐hospital death	2.1%	0.8%	0.177	2.3%	0.863	0.8%	0.010	3.0%	0.352	2.8%	0.347
Discharge disposition			0.194		0.825		<0.001		0.839		0.406
Routine	58.5%	51.0%		60.8%		61.8%		59.9%		54.3%	
Skilled nursing facility	14.8%	21.6%		13.6%		12.1%		13.2%		15.0%	
Home health care	24.0%	26.6%		22.5%		23.9%		23.2%		27.2%	
AKI	12.3%	16.9%	0.069	11.6%	0.705	10.7%	0.178	9.2%	0.077	20.0%	<0.001
AKI leading to dialysis	1.0%	0%	0.205	1.0%	0.957	0.5%	0.158	0.7%	0.559	3.1%	<0.001
PPM implantation	9.9%	9.6%	0.868	9.3%	0.645	9.4%	0.595	14.5%	0.002	12.6%	0.030
Bleeding requiring transfusion	8.3%	18.8%	<0.001	9.2%	0.518	9.5%	0.225	8.0%	0.816	12.4%	<0.001
Stroke/TIA	0.4%	0.5%	0.941	0.4%	0.991	0.3%	0.606	0%	0.253	0.3%	0.615
Length of hospital stay, d	3 (2–6)	3 (2–10)	0.008	3 (2–7)	0.838	3 (2–6)	0.614	2 (2–5)	0.139	3 (2–9)	<0.001
Total inflation‐adjusted cost, US$	$54 770±$31 888	$60 366±$36 596	0.028	$59 452±$35 001	0.010	$53 301±$31 866	0.144	$56 100±$33 509	0.463	$59 610±$34 547	0.001
30‐d readmission rate	13.3%	25.0%	<0.001	15.2%	0.260	15.3%	0.117	10.0%	0.086	17.0%	0.017
Weighted population at 30 d	211 436	381		808		1541		693		970	
Causes of readmission at 30 d											
Congestive heart failure	2.6%	7.9%	<0.001	3.8%	0.049	5.1%	<0.001	2.4%	0.996	3.2%	0.169
Myocardial infarction	0.2%	0.9%	0.009	0%	0.370	0.7%	<0.001	0%	0.409	1.3%	<0.001
Stroke/TIA	0.4%	1.4%	0.039	0.2%	0.469	0.6%	0.572	1.0%	0.082	0.4%	0.754
Bleeding requiring transfusion	0.8%	6.5%	<0.001	5.3%	<0.001	4.9%	<0.001	3.1%	0.021	4.9%	<0.001
90‐d readmission rate	22.5%	48.0%	<0.001	36.1%	<0.001	24.7%	0.064	18.4%	0.023	31.2%	<0.001
Weighted population at 90 d	168 310	298		642		1230		543		756	
Causes of readmission at 90 d											
Congestive heart failure	2.7%	3.4%	0.786	2.3%	0.076	3.1%	0.295	2.2%	0.074	1.9%	0.008
Myocardial infarction	0.3%	0%	0.322	0%	0.146	0.5%	0.332	0%	0.181	0.5%	0.337
Stroke/TIA	0.8%	0.3%	0.327	0.2%	0.053	0.2%	0.019	0.4%	0.215	0%	0.010
Bleeding requiring transfusion	1.1%	9.7%	<0.001	4.8%	<0.001	4.3%	<0.001	1.5%	0.103	5.4%	<0.001
180‐d readmission rate	30.8%	65.1%	<0.001	41.7%	<0.001	31.6%	0.640	24.4%	0.009	40.6%	<0.001
Weighted population at 180 d	107 846	175		372		747		357		507	
Causes of readmission at 180 d											
Congestive heart failure	3.5%	4.0%	0.676	2.4%	0.040	4.3%	0.621	2.2%	0.030	3.7%	0.327
Myocardial infarction	0.5%	0%	0.355	0%	0.178	1.2%	0.005	0%	0.187	0.8%	0.328
Stroke/TIA	1.1%	1.7%	0.543	0.3%	0.097	0.4%	0.043	0.6%	0.261	0.2%	0.037
Bleeding requiring transfusion	1.5%	11.4%	<0.001	6.2%	0.005	5.0%	0.028	2.2%	0.204	6.3%	<0.001

Values are mean±SD, median (interquartile range), or %. AKI indicates acute kidney injury; PPM, permanent pacemaker; and TIA, transient ischemic attack.

Odds ratios of adverse outcomes adjusted for baseline characteristics are shown in the Figure. The presence of active cancer was not significantly associated with in‐hospital mortality after TAVR (adjusted odds ratio, 1.06 [95% CI, 0.89–1.27]; *P*=0.523; Figure [A]). In contrast, the presence of active cancer was significantly associated with increased odds of bleeding requiring transfusion at 30 days and readmission at 30, 90, and 180 days after TAVR (Figure [B] and [D] through [F]). Among individual active cancer types, active colon, prostate, and any type of metastatic cancers were significantly associated with increased odds of bleeding requiring transfusion at 30 days after TAVR (Figure [B]). Active breast and any type of metastatic cancers were significantly associated with PPM implantation at 30 days after TAVR (Figure [C]). Active colon and any type of metastatic cancers were significantly associated with readmission at 30, 90, and 180 days after TAVR (Figure [D] through [F]).

**Figure .**
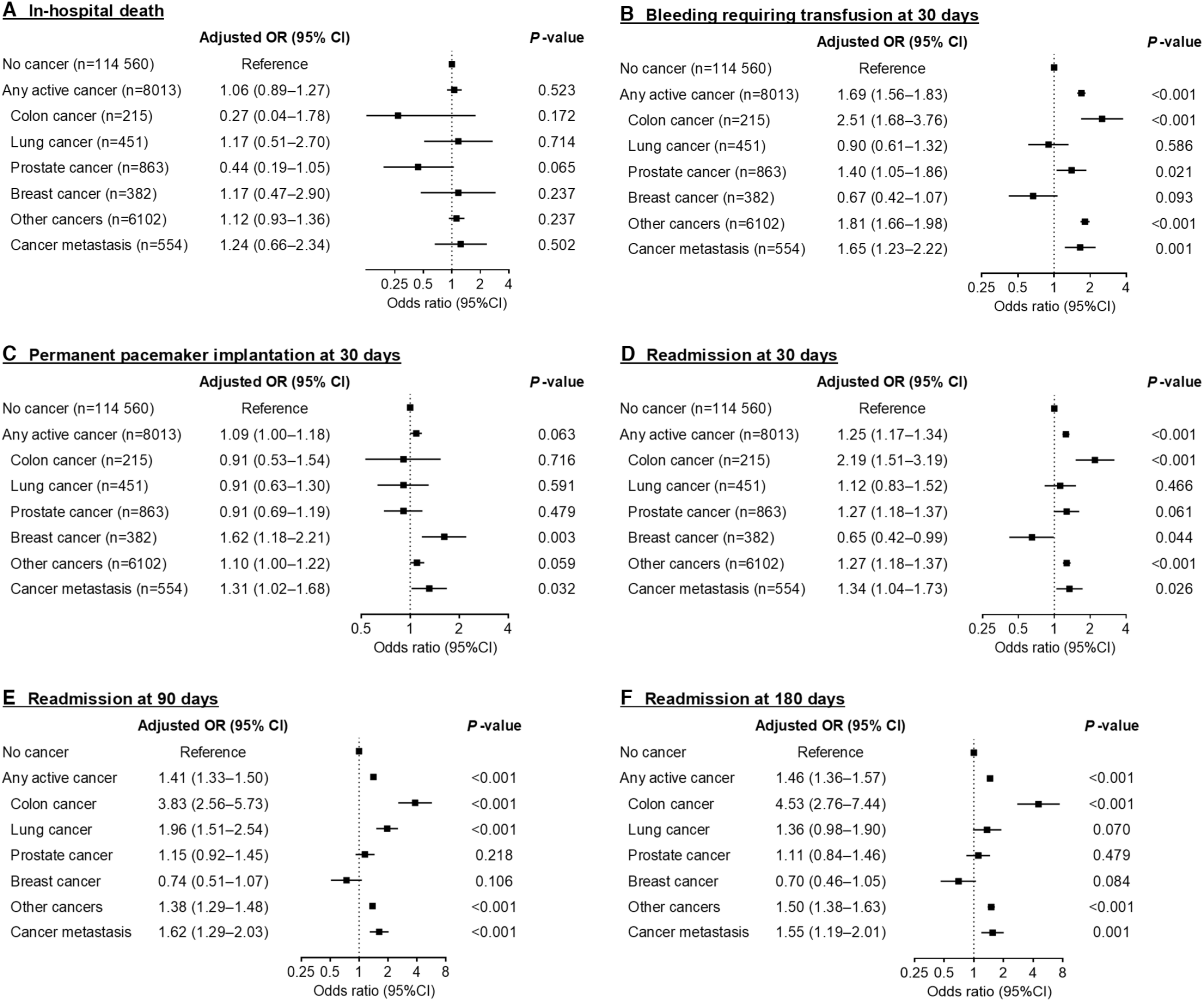
Adjusted ORs for in‐hospital death (A), bleeding requiring transfusion at 30 days (B), permanent pacemaker implantation at 30 days (C), readmission at 30 days (D), readmission at 90 days (E), and readmission at 180 days (F). OR indicates odds ratio.

Table S3 shows the clinical characteristics of the propensity score‐matched cohort. Distributional overlap of propensity score before and after propensity score matching is shown in Figure S1. Absolute standardized mean differences of covariates using propensity score matching is shown in Figure S2. All standardized mean differences were within 0.2, indicating that the propensity score model specification was adequate in this population. Additional information is provided in Data S1.

Among the propensity score‐matched cohort, in‐hospital mortality after TAVR was not significantly different between the 2 groups (2.9% versus 3.0%, *P*=0.650). PPM implantation (11.1% versus 9.9%, *P*=0.016) and bleeding requiring blood transfusion (14.3% versus 10.1%, *P*<0.001) after TAVR were more frequent in patients with active cancer than those without. The rate of readmissions at 30, 90, and 180 days after TAVR were significantly higher in patients with active cancer than those without (Table S4).

## DISCUSSION

We report several important findings in this study: (1) Patients with active cancer who underwent TAVR had similar in‐hospital mortality compared with patients without active cancer. (2) Patients with active cancer were at increased risk of bleeding requiring transfusion and readmission at 30, 90, and 180 days after TAVR compared with those without. (3) Among individual active cancer types, patients with active colon cancer had the highest risks of bleeding requiring transfusion during the index hospitalization and readmission at 30, 90, and 180 days after TAVR. (4) The presence of active metastatic cancer was associated with significantly increased risk of bleeding and readmissions after TAVR. These results suggest the need for an individualized approach to patients with active cancer who undergo TAVR based on the types and extent of cancer, including a pre‐TAVR screening for hemostasis abnormalities, antithrombotic therapy, and postoperative management.

Our findings extend previous observations that patients with active cancer undergoing TAVR have similar in‐hospital mortality compared with those without cancer.^6^, ^7^, ^23^, ^24^ This is clinically important, because it may suggest that TAVR is an appropriate treatment strategy in certain patients with active cancer and severe AS that will allow them to receive first‐line cancer therapy including surgery. Although we did not analyze long‐term mortality in our study, symptomatic severe AS has an average annual mortality of 15% to 25%,^2^ which is a worse prognosis compared with many types and stages of cancer and should be the disease treated first in selected patients. On the other hand, treating these patients with TAVR seems to come along with higher rates of post‐TAVR complications compared with those without active cancer.^6^, ^7^ We used a validated administrative database and enrolled a broad population of patients undergoing TAVR in the United States,^10^, ^12^, ^13^ which may increase the generalizability of the results. The strengths of our study include the large sample size and data on specific cancer types. To our knowledge, this study represents one of the largest focusing on outcomes after TAVR across cancer types.

We observed that patients with active cancer were at increased risk of bleeding requiring transfusion and readmission at 30, 90, and 180 days after TAVR compared with those without. Previous studies demonstrated that patients with active cancer tend to have more bleeding events^6^, ^7^; our study extends beyond that, showing data of in‐hospital complications and readmission rates up to 180 days for individual cancer types. Specifically, patients with active colon cancer had the higher risks of bleeding events after the procedure. This has clinical implications, for example, a no‐antithrombotic strategy may be beneficial for patients with active colon cancer after TAVR, because the recent data revealed the potential benefit of nonantithrombotic therapy for reducing bleeding events without an increase in ischemic events.^25^, ^26^ Another potential explanation for increased risk of readmission may be anemia due to bleeding, cancer, or cancer‐related therapies. Anemia has been found in 30% to 90% of patients with cancer, depending on the type and extent of cancer.^27^ Anemia in patients with cancer can be caused by hemolysis, blood loss, bone marrow failure, infection, and inflammation; all of which are also common side effects of chemotherapy and radiotherapy.^8^, ^27^ Blood disorders, including anemia, are prevalent in patients undergoing TAVR, ranging from 45% to 65%, which have been associated with mortality and bleeding complications after TAVR.^28^ The findings from the PARTNER (Placement of Aortic Transcatheter Valves) I, II, and III trials and registries suggest that moderate‐to‐severe anemia (hemoglobin level<11.0 g/dL) is independently associated with increased 1‐year mortality.^29^ In addition, blood transfusion itself also has been associated with acute kidney injury and long‐term mortality after TAVR, independently of bleeding.^30^ Although the limitations of the data set did not allow us to assess the impact of anemia on outcomes after TAVR in patients with active cancer, further studies are needed to reduce bleeding complications and blood transfusion.

An interesting result of our study was that patients with active breast cancer had a higher risk of PPM insertion after TAVR compared with patients without active cancer. Although we do not have information on left ventricular ejection fraction, and baseline conduction disturbance on electrocardiogram, valve type, or depth of implantation, we might suspect chemotherapy and radiation therapy used for breast cancer may affect conduction abnormality,^31^, ^32^ which predisposes the necessity of a PPM after TAVR. The results suggested that close heart monitor at home after TAVR in patients with active breast cancer may be needed, but that further investigation is needed.

There is no standardized TAVR protocol for patients with active cancer, including the type of valve used, the necessity and duration of antithrombotic therapy, anesthesia during TAVR, and cancer therapies after TAVR. Given the complexity in the management of patients with severe AS and high risks of bleeding complications and readmission after TAVR found in this study, a multidisciplinary cardio‐oncology approach will be needed to optimize these patient outcomes. In general, aortic valve intervention for symptomatic patients with severe AS takes priority over noncardiac surgery, whereas asymptomatic patients with severe AS and normal left ventricular ejection fraction can safely undergo low‐ and intermediate‐risk noncardiac surgery.^33^ The timing of TAVR and noncardiac surgery for malignancy should be individualized based on the specific clinical presentation of each patient. Hospital length and cost of stay were significantly higher in patients with active cancer, especially those with active colon or metastatic cancer, than those without in this study. These trends are similar to those observed in other cardiac catheterization^8^ and surgical procedures,^34^ which are likely to be attributable to bleeding complications. The use of the transradial approach as a secondary access in TAVR may be beneficial to reduce peri‐ and postprocedural bleeding complications and their impact on the cost of care, which can also be applied to transaxillary or transsubclavian TAVR from the contralateral side.

## Study Limitations

The present study has several limitations, and some of which are inherent to the administrative nature of the NRD database. This study did not have information about laboratory data, echocardiographic parameters such as left ventricular ejection fraction, surgical risk scores, implanted valve types (balloon‐expandable versus self‐expandable valve), medications, details of cancer pathogenesis and treatments such as chemotherapy and radiation, and the type of antithrombotic therapy (which is likely to be associated with the incidence of bleeding requiring transfusion after TAVR).^35^, ^36^, ^37^ This study cohort did not include the data of patients undergoing TAVR after 2020, which will allow looking into low‐risk patients undergoing TAVR.^3^, ^4^ However, the mortality and readmission rates after TAVR could be affected by the rapid spread of the COVID‐19 pandemic starting from 2020. The impact of active cancer status on outcomes after TAVR should be further explored using registry data with more comprehensive information on echocardiographic and procedural characteristics, laboratory values, and medications.

## CONCLUSIONS

Patients with active cancer and concomitant AS have similar in‐hospital mortality after TAVR, but with a higher risk of bleeding requiring transfusion and readmission after TAVR, the extent of which depends on the cancer type and the presence of metastasis. These results suggest that TAVR may be a treatment option for certain patients with active cancer and severe AS. An individualized approach to these patients is needed to determine the benefit of TAVR and dictate personalized treatment strategies based on the types and extent of cancers. Long‐term follow‐up data and randomized controlled trials are needed to further investigate this topic.

## Sources of Funding

This work was supported in part by Sakakibara Memorial Research Grant from the Sakakibara Heart Foundation (to Dr Aikawa).

## Disclosures

Dr Kolte has received research funding from the National Institutes of Health/National Heart, Lung, and Blood Institute. Dr Latib is a consultant for and on the advisory board of Medtronic, Abbott, Boston Scientific, and Philips. The remaining authors have no disclosures to report.

## Supporting information

Data S1
Tables S1–S4
Figures S1–S2

## References

[1] Tsao CW , Aday AW , Almarzooq ZI , Alonso A , Beaton AZ , Bittencourt MS , Boehme AK , Buxton AE , Carson AP , Commodore‐Mensah Y , et al. Heart disease and stroke statistics‐2022 update: a report from the American Heart Association. Circulation. 2022;145:e153–e639. doi: 10.1161/CIR.0000000000001052 35078371

[2] Otto CM , Nishimura RA , Bonow RO , Carabello BA , Erwin JP III , Gentile F , Jneid H , Krieger EV , Mack M , McLeod C , et al. 2020 ACC/AHA guideline for the management of patients with valvular heart disease: a report of the American College of Cardiology/American Heart Association Joint Committee on Clinical Practice Guidelines. Circulation. 2021;143:e72–e227. doi: 10.1161/CIR.0000000000000923 33332150

[3] Mack MJ , Leon MB , Thourani VH , Makkar R , Kodali SK , Russo M , Kapadia SR , Malaisrie SC , Cohen DJ , Pibarot P , et al. Transcatheter aortic‐valve replacement with a balloon‐expandable valve in low‐risk patients. N Engl J Med. 2019;380:1695–1705. doi: 10.1056/NEJMoa1814052 30883058

[4] Popma JJ , Deeb GM , Yakubov SJ , Mumtaz M , Gada H , O'Hair D , Bajwa T , Heiser JC , Merhi W , Kleiman NS , et al. Transcatheter aortic‐valve replacement with a self‐expanding valve in low‐risk patients. N Engl J Med. 2019;380:1706–1715. doi: 10.1056/NEJMoa1816885 30883053

[5] Guha A , Dey AK , Arora S , Cavender MA , Vavalle JP , Sabik JF III , Jimenez E , Jneid H , Addison D . Contemporary trends and outcomes of percutaneous and surgical aortic valve replacement in patients with cancer. J Am Heart Assoc. 2020;9:e014248. doi: 10.1161/JAHA.119.014248 31960751 PMC7033818

[6] Landes U , Iakobishvili Z , Vronsky D , Zusman O , Barsheshet A , Jaffe R , Jubran A , Yoon SH , Makkar RR , Taramasso M , et al. Transcatheter aortic valve replacement in oncology patients with severe aortic stenosis. JACC Cardiovasc Interv. 2019;12:78–86. doi: 10.1016/j.jcin.2018.10.026 30621982

[7] Jain V , Saad AM , Gad MM , Bansal A , Abdelfattah O , Farwati M , Ahuja KR , Yun J , Krishnaswamy A , Kapadia SR . Outcomes of cancer patients undergoing transcatheter aortic valve replacement. JACC CardioOncol. 2020;2:506–508. doi: 10.1016/j.jaccao.2020.05.023 34396258 PMC8352026

[8] Kwok CS , Wong CW , Kontopantelis E , Barac A , Brown SA , Velagapudi P , Hilliard AA , Bharadwaj AS , Chadi Alraies M , Mohamed M , et al. Percutaneous coronary intervention in patients with cancer and readmissions within 90 days for acute myocardial infarction and bleeding in the USA. Eur Heart J. 2021;42:1019–1034. doi: 10.1093/eurheartj/ehaa1032 33681960

[9] Bendary A , Ramzy A , Bendary M , Salem M . Transcatheter aortic valve replacement in patients with severe aortic stenosis and active cancer: a systematic review and meta‐analysis. Open Heart. 2020;7:e001131. doi: 10.1136/openhrt-2019-001131 32201582 PMC7066604

[10] Ando T , Onishi T , Kuno T , Briasoulis A , Takagi H , Grines CL , Hatori K , Tobaru T , Malik AH , Ahmad H . Transcatheter versus surgical aortic valve replacement in the United States (from the Nationwide Readmission Database). Am J Cardiol. 2021;148:110–115. doi: 10.1016/j.amjcard.2021.02.031 33667440

[11] Morita S , Malik AH , Kuno T , Ando T , Kaul R , Yandrapalli S , Briasoulis A . Analysis of outcome of 6‐month readmissions after percutaneous left atrial appendage occlusion. Heart. 2022;108:606–612. doi: 10.1136/heartjnl-2021-319345 34400473

[12] Aikawa T , Kuno T , Malik AH , Briasoulis A , Latib A . Short‐term outcomes after transcatheter aortic valve implantation with or without amyloidosis 2012 to 2019. Am J Cardiol. 2022;169:149–151. doi: 10.1016/j.amjcard.2022.01.009 35193762

[13] Ando T , Ashraf S , Kuno T , Briasoulis A , Takagi H , Grines C , Malik A . Hospital variation of 30‐day readmission rate following transcatheter aortic valve implantation. Heart. 2022;108:219–224. doi: 10.1136/heartjnl-2020-318583 33627399

[14] Kawamura I , Kuno T , Sahashi Y , Tanaka Y , Passman R , Briasoulis A , Malik AH . Thirty‐day readmission rate of same‐day discharge protocol after left atrial appendage occlusion: a propensity score‐matched analysis from the National Readmission Database. Heart Rhythm. 2022;19:1819–1825. doi: 10.1016/j.hrthm.2022.07.006 35835364

[15] Kishino Y , Kuno T , Malik AH , Lanier GM , Sims DB , Ruiz Duque E , Briasoulis A . Effect of pulmonary artery pressure‐guided therapy on heart failure readmission in a nationally representative cohort. ESC Heart Fail. 2022;9:2511–2517. doi: 10.1002/ehf2.13956 35560987 PMC9288808

[16] Sahashi Y , Kuno T , Tanaka Y , Passman R , Briasoulis A , Malik AH . The 30‐day readmission rate of same‐day discharge protocol following catheter ablation for atrial fibrillation: a propensity score‐matched analysis from National Readmission Database. Europace. 2022;24:755–761. doi: 10.1093/europace/euab296 34904164

[17] Aikawa T , Kuno T , Van den Eynde J , Briasoulis A , Malik AH . Effects of clinical trial or research program participation status on in‐hospital mortality after transcatheter aortic valve implantation. J Am Heart Assoc. 2022;11:e025920. doi: 10.1161/JAHA.121.025920 35699177 PMC9238665

[18] Grant JK , Vincent L , Ebner B , Maning J , Singh H , Olorunfemi O , Olarte NI , Colombo R , Lopes G , Braghiroli J . In‐hospital outcomes in patients with a history of malignancy undergoing transcatheter aortic valve implantation. Am J Cardiol. 2021;142:109–115. doi: 10.1016/j.amjcard.2020.11.029 33285093

[19] Siegel RL , Miller KD , Fuchs HE , Jemal A . Cancer statistics, 2022. CA Cancer J Clin. 2022;72:7–33. doi: 10.3322/caac.21708 35020204

[20] Quan H , Sundararajan V , Halfon P , Fong A , Burnand B , Luthi JC , Saunders LD , Beck CA , Feasby TE , Ghali WA . Coding algorithms for defining comorbidities in ICD‐9‐CM and ICD‐10 administrative data. Med Care. 2005;43:1130–1139. doi: 10.1097/01.mlr.0000182534.19832.83 16224307

[21] Khera R , Angraal S , Couch T , Welsh JW , Nallamothu BK , Girotra S , Chan PS , Krumholz HM . Adherence to methodological standards in research using the National Inpatient Sample. JAMA. 2017;318:2011–2018. doi: 10.1001/jama.2017.17653 29183077 PMC5742631

[22] Yokoyama Y , Kuno T , Malik A , Briasoulis A . Outcomes of robotic coronary artery bypass versus nonrobotic coronary artery bypass. J Card Surg. 2021;36:3187–3192. doi: 10.1111/jocs.15710 34091953

[23] Watanabe Y , Kozuma K , Hioki H , Kawashima H , Nara Y , Kataoka A , Shirai S , Tada N , Araki M , Takagi K , et al. Comparison of results of transcatheter aortic valve implantation in patients with versus without active cancer. Am J Cardiol. 2016;118:572–577. doi: 10.1016/j.amjcard.2016.05.052 27324159

[24] Mangner N , Woitek FJ , Haussig S , Holzhey D , Stachel G , Schlotter F , Höllriegel R , Mohr FW , Schuler G , Linke A . Impact of active cancer disease on the outcome of patients undergoing transcatheter aortic valve replacement. J Interv Cardiol. 2018;31:188–196. doi: 10.1111/joic.12458 29166702

[25] Kobari Y , Inohara T , Tsuruta H , Yashima F , Shimizu H , Fukuda K , Naganuma T , Mizutani K , Yamawaki M , Tada N , et al. No antithrombotic therapy after transcatheter aortic valve replacement: insight from the OCEAN‐TAVI registry. JACC Cardiovasc Interv. 2023;16:79–91. doi: 10.1016/j.jcin.2022.10.010 36599591

[26] Kobari Y , Inohara T , Hayashida K . Antithrombotic therapy after transcatheter aortic valve replacement. Cardiovasc Interv Ther. 2023;38:9–17. doi: 10.1007/s12928-022-00893-9 36115004

[27] Knight K , Wade S , Balducci L . Prevalence and outcomes of anemia in cancer: a systematic review of the literature. Am J Med. 2004;116(Suppl 7A):11s–26s. doi: 10.1016/j.amjmed.2003.12.008 15050883

[28] De Larochellière H , Puri R , Eikelboom JW , Rodés‐Cabau J . Blood disorders in patients undergoing transcatheter aortic valve replacement: a review. JACC Cardiovasc Interv. 2019;12:1–11. doi: 10.1016/j.jcin.2018.09.041 30621965

[29] Bhardwaj B , Kolte D , Zhao Y , Alu M , Zahr F , Passeri J , Inglessis I , Vlahakes G , Garcia S , Cohen D , et al. Severity of and recovery from anemia after transcatheter aortic valve replacement: an analysis of the PARTNER trials and registries. J Soc Cardiovasc Angiogr Interv. 2023;2:100531. doi: 10.1016/j.jscai.2022.100531 PMC11307816 39132543

[30] Nuis RJ , Rodés‐Cabau J , Sinning JM , van Garsse L , Kefer J , Bosmans J , Dager AE , van Mieghem N , Urena M , Nickenig G , et al. Blood transfusion and the risk of acute kidney injury after transcatheter aortic valve implantation. Circ Cardiovasc Interv. 2012;5:680–688. doi: 10.1161/CIRCINTERVENTIONS.112.971291 23048055

[31] Schmitz KH , Prosnitz RG , Schwartz AL , Carver JR . Prospective surveillance and management of cardiac toxicity and health in breast cancer survivors. Cancer. 2012;118:2270–2276. doi: 10.1002/cncr.27462 22488701

[32] Errahmani MY , Thariat J , Ferrières J , Panh L , Locquet M , Lapeyre‐Mestre M , Guernec G , Bernier MO , Boveda S , Jacob S . Risk of pacemaker implantation after radiotherapy for breast cancer: a study based on French nationwide health care database sample. Int J Cardiol Heart Vasc. 2022;38:100936. doi: 10.1016/j.ijcha.2021.100936 35005214 PMC8717594

[33] Halvorsen S , Mehilli J , Cassese S , Hall TS , Abdelhamid M , Barbato E , De Hert S , de Laval I , Geisler T , Hinterbuchner L , et al. 2022 ESC Guidelines on cardiovascular assessment and management of patients undergoing non‐cardiac surgery. Eur Heart J. 2022;43:3826–3924. doi: 10.1093/eurheartj/ehac270 36017553

[34] Guha A , Dey AK , Kalra A , Gumina R , Lustberg M , Lavie CJ , Sabik JF III , Addison D . Coronary artery bypass grafting in cancer patients: prevalence and outcomes in the United States. Mayo Clin Proc. 2020;95:1865–1876. doi: 10.1016/j.mayocp.2020.05.044 32861331 PMC7860624

[35] Dangas GD , Tijssen JGP , Wöhrle J , Søndergaard L , Gilard M , Möllmann H , Makkar RR , Herrmann HC , Giustino G , Baldus S , et al. A controlled trial of rivaroxaban after transcatheter aortic‐valve replacement. N Engl J Med. 2020;382:120–129. doi: 10.1056/NEJMoa1911425 31733180

[36] Van Mieghem NM , Unverdorben M , Hengstenberg C , Möllmann H , Mehran R , López‐Otero D , Nombela‐Franco L , Moreno R , Nordbeck P , Thiele H , et al. Edoxaban versus vitamin K antagonist for atrial fibrillation after TAVR. N Engl J Med. 2021;385:2150–2160. doi: 10.1056/NEJMoa2111016 34449183

[37] Calabrò P , Gragnano F , Niccoli G , Marcucci R , Zimarino M , Spaccarotella C , Renda G , Patti G , Andò G , Moscarella E , et al. Antithrombotic therapy in patients undergoing transcatheter interventions for structural heart disease. Circulation. 2021;144:1323–1343. doi: 10.1161/CIRCULATIONAHA.121.054305 34662163

