# Supplementary material for: Transcatheter Aortic Valve Replacement in Patients With or Without Active Cancer
Source: J Am Heart Assoc. 2023 Oct 27;12(21):e030072. doi: 10.1161/JAHA.123.030072 (PMC10727376; doi:10.1161/JAHA.123.030072)

Supplemental Material

Data S1.

SUPPLEMENTAL METHODS

R code for the propensity score matching

The propensity score matching was performed using the following R code.

```
> m.outtavrcancer<-
matchit(neo~discwt+year+age+female+chfs+htn+dm+obese+pvds+cad+afib+pulmis+pulm+
otherneuro+lowtsh+ckdesrd+liver+anemia+rheum+coagul+wtloss+oldcabg+oldmi+oldpci+ol
dstroke+hosp_bedsize+hosp_ur_teach+pay1+zipinc_qrtl, caliper=0.2,
data=tavr_cancer_prematch)
> m.datatavrcancer30<-match.data(m.outtavrcancer)
> write.csv(m.datatavrcancer30, "m.datatavrcancer30.csv")
```

Table S1. ICD-9 and ICD-10 clinical classifications software (CCS) codes corresponding to cancer types. ICD-9 and ICD-10 share the same CCS codes.

Single-level CCS diagnosis code	Cancer type
11	Cancer of head and neck
12	Cancer of esophagus
13	Cancer of stomach
14	Cancer of colon
15	Cancer of rectum and anus
16	Cancer of liver and intrahepatic bile duct
17	Cancer of pancreas
18	Cancer of other GI organs; peritoneum
19	Cancer of bronchus; lung
20	Cancer, other respiratory and intrathoracic
21	Cancer of bone and connective tissue
22	Melanomas of skin
23	Other non-epithelial cancer of skin
24	Cancer of breast
25	Cancer of uterus
26	Cancer of cervix
27	Cancer of ovary
28	Cancer of other female genital organs
29	Cancer of prostate
30	Cancer of testis
31	Cancer of other male genital organs
32	Cancer of bladder
33	Cancer of kidney and renal pelvis
34	Cancer of other urinary organs
35	Cancer of brain and nervous system
36	Cancer of thyroid
37	Hodgkin's disease
38	Non-Hodgkin's lymphoma
39	Leukemias
40	Multiple myeloma
41	Cancer, other and unspecified primary

Table S2. ICD-9 and ICD-10 codes used for the identification of events.

Events	ICD-9 codes	ICD-10 codes
AKI	584*	N17*
AKI leading to dialysis	AKI code along with 3995*, 5498*	AKI code along with 3E1M39Z*, 5A1D*
PPM implantation	0050*, 3780*, 3781*, 3782*, 3783*	02HK3JZ, 02H60JZ, 02H60NZ, 02H63JZ, 02H63NZ, 02H64JZ, 02H64NZ, 02HK0JZ, 02HK0NZ, 02HK3JZ, 02HK3NZ, 02HK4JZ, 02HK4NZ, 02HN0JZ, 02HN4JZ, 0JH604Z, 0JH605Z, 0JH606Z, 0JH607Z, 0JH634Z, 0JH635Z, 0JH636Z, 0JH637Z
Congestive heart failure	398.91, 402.01, 402.11, 402.91, 404.01, 404.03, 404.11, 404.13, 404.91, 404.93, 425.4–425.9, 428.*	I09.9, I11.0, I13.0, I13.2, I25.5, I42.0, I42.5–I42.9, I43.*, I50.*, P29.0
Myocardial infarction	410.*	I21.*, I22.*
Stroke	431, 433.*1, 434.*1, 435*, 433.6*, 997.01	I63, I63.0, I63.1, I63.2, I63.3, I63.4, I63.5, I63.6, I63.8, I63.9, I64
TIA	435*	G45, G45.0, G45.1, G45.2, G45.3, G45.4, G45.8, G45.9
Major bleeding	431*, 432.9, 459.0*, 578.9, 568.81, V582*, 99.00	I61*, I62*, I69*, I85*, I92*, K22*, K25*, K26*, K27*, K29*, K50*, K51*, K57*, K62*, K92*
Blood transfusion	99.0, 99.01, 99.02, 99.03, 99.04	30233H0, 30233H1, 30233N0, 30233N1, 30233P1, 30233W0, 30243H0, 30243H1, 30243N0, 30243N1, 30243P1, 30243W0, 30253H0, 30253H1, 30253N0, 30253N1, 30253P, 30253W0, 30263H0, 30263H1, 30263N0, 30263N1, 30263P1, 30263W0

Table S3. Comparisons of clinical characteristics between the propensity score-matched groups, with a caliper width equal to 0.2 of the standard deviation of the propensity score.

Characteristics	No cancer (n=8,012)	Any cancer (n=8,012)	P value
Weighted population	14,720	14,720	
Age, y	79.4 ± 9.1	79.6 ± 8.4	0.303
Median (interquartile range)	81 (74-86)	81 (75-86)	0.523
Women	40.8%	41.2%	0.708
Insurance			0.744
Medicare	89.2%	89.5%	
Medicaid	1.4%	1.2%	
Private	6.7%	6.9%	
Other	2.7%	2.4%	
Median household income			0.035
$1-$38,999	18.9%	18.7%	
$39,000-$47,999	23.8%	25.8%	
$48,000-$62,999	28.9%	27.3%	
$63,000 or more	28.4%	28.1%	
**Comorbidities**			
Congestive heart failure	77.1%	76.8%	0.739
Hypertension	84.5%	84.1%	0.570
Diabetes	35.5%	34.8%	0.383
Obesity	15.6%	16.3%	0.283
Peripheral vascular disease	24.2%	23.0%	0.116
Coronary artery disease	66.9%	66.7%	0.766
Atrial fibrillation	41.1%	40.6%	0.633
Chronic pulmonary disease	34.2%	34.6%	0.564
Pulmonary circulation disorders	21.5%	21.9%	0.585
Other neurological disorders	6.5%	6.3%	0.575
Hypothyroidism	19.0%	19.8%	0.212
CKD without end-stage renal disease	39.0%	39.0%	0.953
Liver disease	5.5%	5.5%	0.931
Anemia	6.9%	7.3%	0.369
Rheumatic disease	5.0%	4.9%	0.617
Coagulopathy	21.0%	20.5%	0.434
Abnormal weight loss	6.1%	6.4%	0.545
Prior CABG	14.9%	14.7%	0.707
Prior myocardial infarction	10.2%	10.3%	0.830
Prior PCI	18.5%	18.4%	0.799
Prior stroke	11.1%	11.3%	0.826
**Hospital characteristics**			
Hospital bed size			0.409
Small	4.4%	4.0%	
Medium	18.9%	18.8%	
Large	76.7%	77.3%	
Hospital location/teaching status			0.051
Rural teaching	9.9%	9.4%	
Urban teaching	89.1%	89.8%	
Non-teaching	1.0%	0.8%	

Values are mean ± standard deviation, or %. AKI = acute kidney injury; CABG = coronary artery bypass grafting; CKD = chronic kidney disease; PCI = percutaneous coronary intervention.

Table S4. Comparisons of clinical outcomes after TAVR between the propensity score-matched groups, with a caliper width equal to 0.2 of the standard deviation of the propensity score.

Characteristics	No cancer (n=8,012)	Any cancer (n=8,012)	P value
**In-hospital death**	3.0%	2.9%	0.650
Discharge disposition			0.128
Routine	55.4%	53.3%	
Skilled nursing facility	16.6%	16.8%	
Home health care	24.3%	26.4%	
AKI	14.7%	16.7%	0.001
AKI leading to dialysis	1.4%	1.9%	0.079
PPM implantation	9.9%	11.1%	0.016
Bleeding requiring transfusion	10.1%	14.3%	<0.001
Stroke/TIA	0.6%	0.6%	0.998
Total inflation-adjusted cost, US dollars	\$58,304 ± \$34,916	\$61,533 ± \$40,266	<0.001
**30-day readmission rate**	14.1%	17.1%	<0.001
Causes of readmission at 30 days			
Congestive heart failure	2.4%	3.0%	0.009
Myocardial infarction	0.2%	0.2%	0.894
Stroke/TIA	0.4%	0.4%	0.738
Bleeding requiring transfusion	2.0%	3.2%	<0.001
**90-day readmission rate**	22.5%	29.1%	<0.001
Causes of readmission at 90 days			
Congestive heart failure	2.4%	2.7%	0.094
Myocardial infarction	0.4%	0.3%	0.266
Stroke/TIA	0.8%	0.6%	0.070
Bleeding requiring transfusion	1.4%	2.4%	<0.001
**180-day readmission rate**	27.0%	45.1%	<0.001
Causes of readmission at 180 days			
Congestive heart failure	2.8%	4.2%	<0.001
Myocardial infarction	0.3%	0.5%	0.062
Stroke/TIA	0.7%	0.9%	0.107
Bleeding requiring transfusion	2.6%	3.7%	<0.001

Values are mean ± standard deviation, or %. AKI = acute kidney injury; PPM = permanent pacemaker; TIA = transient ischemic attack.

Figure S1. Distributional overlap of propensity score before and after propensity score matching.

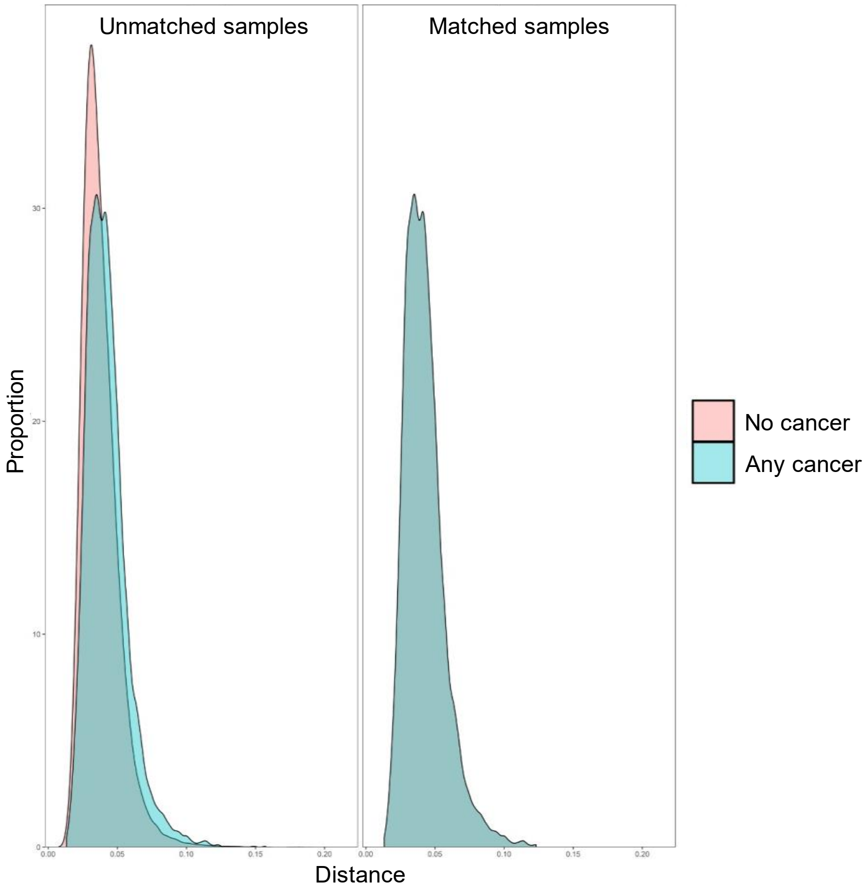

Figure S2. Absolute standardized mean differences of covariates before and after propensity score matching.

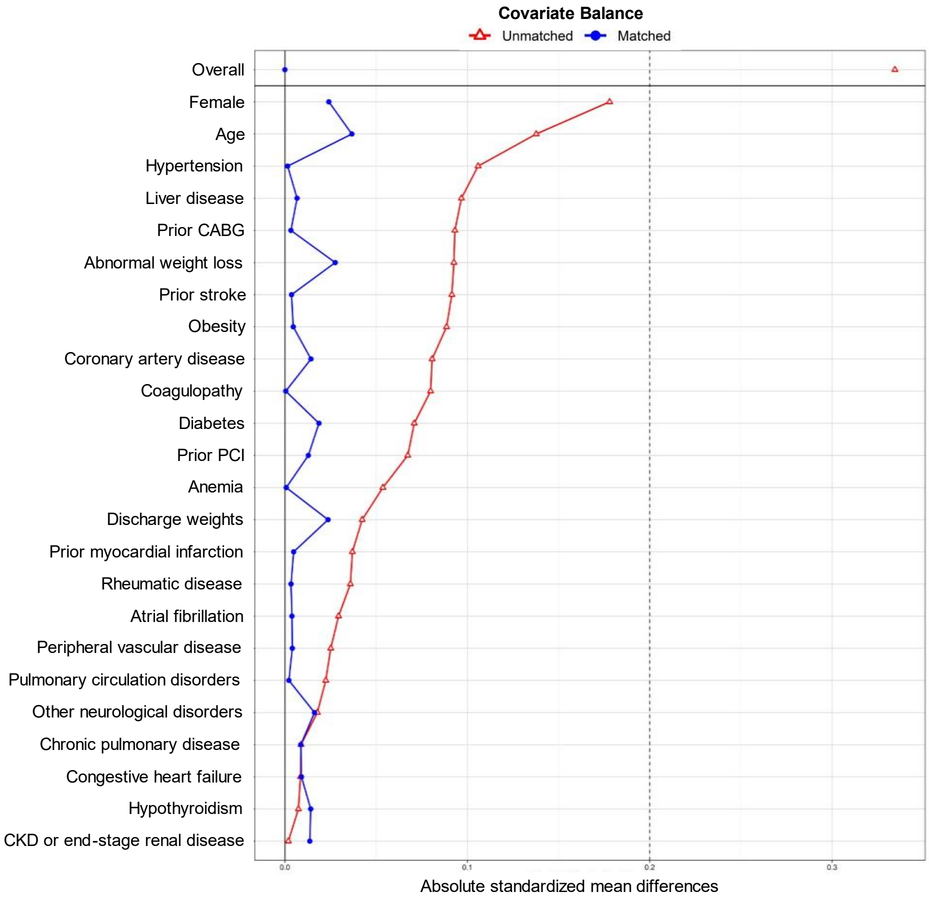

Supplement: Supplementary file 1 — Data S1 Tables S1–S4 Figures S1–S2 [file JAH3-12-e030072-s001.pdf]
